# Comparison of HyFoSy, HyCoSy and X-Ray Hysterosalpingography in the Assessment of Tubal Patency in Women with Infertility: A Systematic Review and Meta-Analysis

**DOI:** 10.3390/medsci13030168

**Published:** 2025-09-02

**Authors:** Emmanouil M. Xydias, Vasileios Emmanouil, Maria Koutini, Anna Ntanika, Elias Tsakos, Matthew Prior, Ippokratis Sarris, Ioannis Thanasas, Alexandros Daponte, Apostolos C. Ziogas

**Affiliations:** 1 EmbryoClinic IVF, 6 Andrianoupoleos, 55133 Thessaloniki, Greece; 2 School of Health Sciences, Faculty of Medicine, Aristotle University of Thessaloniki, AUTH Campus, 54124 Thessaloniki, Greece; 3 Faculty of Health Sciences, Department of Medicine, Democritus University of Thrace, Alexandroupoli Campus, 68100 Alexandroupoli, Greece; 4 School of Health Sciences, Faculty of Medicine, University of Ioannina, Doroutis Campus, 45110 Ioannina, Greece; 5 Newcastle Fertility Centre, International Centre for Life, Department of Reproductive Medicine, Times Square, Newcastle upon Tyne NE1 4EP, UK; 6 King’s Fertility Centre, The Fetal Medicine Research Institute, 16-20 Windsor Walk Denmark Hill, London SE5 8BB, UK; 7 Faculty of Life Sciences & Medicine, Department of Women’s and Children’s Health, King’s College, Strand Campus, London WC2R 2LS, UK; 8 Department of Obstetrics and Gynecology, General Hospital of Trikala, 56 Karditsis, 42131 Trikala, Greece; 9 School of Health Sciences, Faculty of Medicine, University of Thessaly, 3 Panepistimiou, 41500 Larissa, Greece

**Keywords:** HyFoSy, HyCoSy, hysterosalpingography, tubal patency, diagnostic accuracy, pain tolerability

## Abstract

**Background/Objectives**: Tubal dysfunction may be a contributing factor in up to 35% of infertility cases, rendering tubal patency assessment a vital component of the infertility workup. In this review we examined the diagnostic efficacy and tolerability of hysterosalpingo-foam sonography (HyFoSy) and compared it to hysterosalpingography (HSG) and hysterosalpingo-contrast sonography (HyCoSy). **Methods**: Online databases were systematically searched and evaluated according to the PRISMA 2020 guidelines. Statistical heterogeneity was assessed. Diagnostic sensitivity, specificity and inter-method agreement were evaluated, along with mean pain scores. **Results**: This analysis included data from 9 studies and 1354 patients with conclusive diagnostic data from 2422 tubes and 1294 patients with data on intra-procedural pain. With regard to HyFoSy and HyCoSy comparison, pooled sensitivity was 87% and 69%, respectively (*p* = 0.074), while pooled specificity was 95% and 85%, respectively, favoring HyFoSy (*p* < 0.001). HyFoSy was more tolerable with regard to pain, but this was not statistically significant. Regarding the HyFoSy and HSG comparison, pooled Cohen’s k was 0.38, indicating fair-moderate agreement. In subsequent analysis, with HSG as a reference standard, HyFoSy demonstrated low sensitivity (61%) but high specificity (87%). With regard to experienced pain, HyFoSy and HSG had a difference of 2.4 units on a 10-point scale, favoring HyFoSy (*p* < 0.001). **Conclusions**: HyFoSy was superior to HyCoSy and may be used as a first-line tubal assessment method, with HSG being utilized in inconclusive cases. However, further research is still required due to the small number of available studies.

## 1. Introduction

Infertility is estimated to affect up to 12% of couples worldwide [1,2], with tubal factor infertility in particular being a significant contributing factor in over 35% of all infertility cases [3]. Tubal factor infertility may be caused by a wide range of structural abnormalities of the fallopian tubes, such as occlusion, stenosis or dilation, as well as impaired peristaltic function [4,5].

Given the increased incidence of tubal factor infertility, the assessment of tubal patency as a part of standard infertility workup is a crucial step, necessitating the use of a safe, easily conducted, tolerable and inexpensive diagnostic method. To date, the most commonly used methods are hysterosalpingography (HSG) and hysterosalpingo-contrast sonography (HyCoSy) [6]. However, both methods are associated with certain disadvantages. In particular, HSG, while possessing remarkable diagnostic accuracy, has been known to cause discomfort, pain and adverse reactions [7] and necessitates exposure to ionizing radiation. HyCoSy utilizes a saline-based contrast agent and is more tolerable, with fewer adverse reactions and no exposure to radiation. However, as far as diagnostic accuracy is concerned, while some studies have demonstrated that it is an efficacious diagnostic modality [8,9], there have been those that have reported it to be severely lacking in diagnostic accuracy compared with other methods [10].

The newest available method of tubal patency assessment is hysterosalpingo-foam sonography (HyFoSy). HyFoSy is an ultrasonographic technique that utilizes a foam-based contrast agent, consisting of a solution of hydroxyethylcellulose and glycerol gel (ExEm^®^ gel), which results in a more stable medium with extended echogenicity, theoretically ensuring superior imaging capabilities [11,12]. The aim of the present systematic review and meta-analysis is to evaluate the diagnostic efficacy of HyFoSy in comparison to the traditionally used methods of HSG and HyCoSy. The findings of the present review were presented at the ESHRE 2023 Conference and later published as an abstract [13].

## 2. Materials and Methods

### 2.1. Protocol and Registration

The systematic review was conducted according to the Preferred Reporting Items for Systematic Reviews and Meta-Analyses (PRISMA 2020) recommendations [14]. The application of the PRISMA 2020 criteria ensures that the improved transparency of the methodology was adhered to for the completion of this review, as it follows a structured format, in addition to better consistency with the rest of the literature, facilitating comparison of findings. Furthermore, adherence to these guidelines improves the replicability of the findings, leading to more reliable and objective outcomes [14]. A complete research protocol was drafted and registered in the PROSPERO database for systematic reviews, available under Protocol ID: CRD42022343545.

### 2.2. Eligibility Criteria

The rationale for the systematic review question was based on the PICOS (patient or population/index test/comparator/outcomes/study design) framework, recommended by the Cochrane Collaboration [15]. Studies on adult women diagnosed with infertility were included while studies on immunosuppressed women or women who have undergone sterilization procedures (tubal coil placement, tubal ligation, etc.) were considered ineligible. The index test was HyFoSy. The comparator tests were HyCoSy and HSG. The primary outcome was the diagnostic performance of the tests and the secondary outcome was the patient-reported pain during the diagnostic procedure. Regarding the study design, only primary, published studies were included. Secondary studies like systematic or literature reviews, as well as published abstracts of conference proceedings and reports of individual cases, were excluded. Studies on animal or cadaveric models were excluded as well.

### 2.3. Information Sources and Search Strategy

The studies selected for this systematic review were retrieved from MEDLINE/PubMed, Web of Science, Scopus, Ovid and CINAHL databases, which were systematically searched until March 2025. Additional studies were sought via citation searching of relevant reviews, so as not to omit any relevant reports that might have been missed during the systematic search; however, no additional eligible studies were found. The search sequence used in all three databases was “(Hysterosalpingo* AND (foam OR contrast) AND (hyfosy OR hycosy)”. No other filters or limitations were applied during the process.

### 2.4. Selection Process

The studies retrieved from the systematic search described above were screened by four authors independently. No automated tools were used in the screening process. Duplicate records and irrelevant records were removed manually. Any resulting inconsistencies between the three independently working authors were resolved by the input of a fourth.

### 2.5. Data Collection Process

The data extraction from every eligible study was conducted by two authors independently and one author who revised the results. The data that were sought and extracted from each study were about study design (study period, type of study, total sample size, year of publication), patients’ characteristics (mean age, mean infertility duration), comparator and reference test features, and technical characteristics (US system model, contrast medium, applied frequency, etc.).

### 2.6. Data Items

Data extracted regarding primary outcomes were true positive, false positive, false negative and true negative number of patients, for the studies where a reference test was used, and thus sensitivity and specificity could be calculated. Tubal obstruction was defined as the positive result and tubal patency as the negative result. For studies that assessed inter-method agreement, raw data on the number of patients with method agreement and disagreement were extracted and Cohen’s k values were calculated. Results that were labeled as inconclusive by the investigators or cases of test failure were excluded from the data synthesis. From the available patient data, the number of tubes assessed was extracted or extrapolated using provided information. Therefore, in this analysis, all diagnostic accuracy or agreement outcomes were calculated based on the status of individual tubes. This methodology was preferred in order to classify results in a binary format (obstructed/patent) instead of including multiple categories (both tubes obstructed/patent, right tube patent/left tube obstructed, right tube obstructed, left removed, etc.). Outcomes regarding intra-procedural pain were calculated per individual patient.

### 2.7. Risk of Bias Assessment

Risk of bias was assessed for each included study using suitable assessment tools. For diagnostic accuracy studies, the QUADAS-2C tool (4 September 2021 version) [16] was used for the assessment of the applicability and risk of bias, which was also indicated by the Cochrane Handbook for diagnostic test accuracy reviews [15]. Regarding rating, if every signaling question in a domain had a positive answer, that domain was rated as “Low Risk” and if it had a negative answer, it was rated as “High Risk”. If no clear answer could be extracted, the domain was rated “Unclear”, depending on the individual circumstances of each study. The overall rating was derived from the rating of the majority of the domains. For non-diagnostic, randomized studies, the RoB 2 tool (22 August 2019 version) [17] was used, as also recommended by the Cochrane Handbook (Version 1.0.0, 2009) [15]. For non-diagnostic, non-randomized studies, the Newcastle–Ottawa Scale (3 May 2021 version) was utilized [18]. The risk of bias assessment was conducted by two authors independently and inconsistencies were resolved by the input of a third author.

### 2.8. Statistical Analysis

Statistical heterogeneity between the studies was evaluated with the use of Cochran’s Q and the I2 statistic, as formally suggested in the Cochrane Handbook for systematic reviews [15]. A *p* value less than 0.05 was considered statistically significant and I2 < 50% was considered non-significant heterogeneity. In the studies where a reference standard was used, pooled percentages of sensitivity and specificity were calculated and compared using relative diagnostic parameters. Regarding the comparison, a *p* value of less than 0.05 was considered a statistically significant difference. Results were presented in forest plots. In the studies where no reference standard was used, Cohen’s k was calculated to assess agreement between the methods used. A pooled kappa statistic was calculated and agreement was evaluated as follows: k ≤ 0 indicated no agreement, 0.01–0.20 none to slight, 0.21–0.40 fair, 0.41–0.60 moderate, 0.61–0.80 substantial and 0.81–1.00 as almost perfect agreement [19]. Calculations and graphs were carried out using the Stata Statistical Software: Release 14.2 by StataCorp LP.

## 3. Results

**Figure 1:**
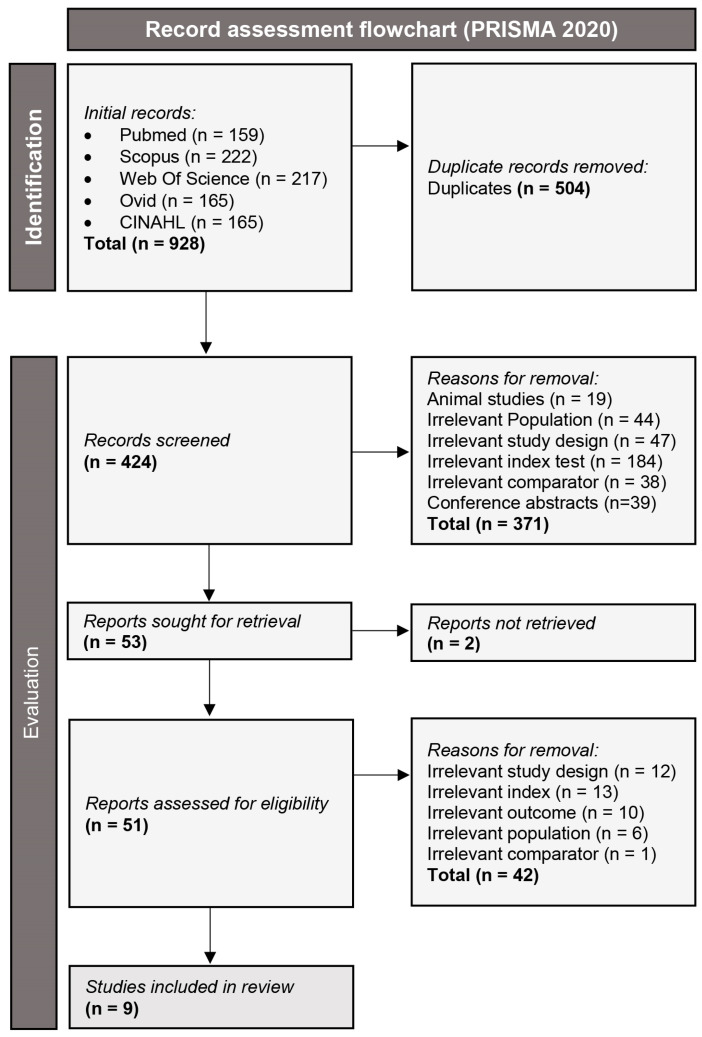
Flowchart of the applied study assessment process, as specified by the PRISMA 2020 guidelines.

The initial search of the databases yielded a pool of 928 studies, which were rigorously evaluated in a two-step process, in accordance with the PRISMA 2020 algorithm. Ultimately, nine studies were eligible for inclusion in this study [20,21,22,23,24,25,26,27,28]. This process is summarized in detail in Figure 1. Of the included studies, with regard to diagnostic efficacy there were two diagnostic accuracy studies comparing HyFoSy and HyCoSy and using laparoscopic chromopertubation as a reference standard [24,25], one inter-method agreement study comparing HyFoSy and HyCoSy [23] and four inter-method agreement studies comparing HyFoSy and HSG [20,21,26,27]. With regard to intra-procedural pain experienced by the patient, one study comparing HyFoSy and HyCoSy [23] and three studies comparing HyFoSy and HSG [22,27,28]. The eligible studies cumulatively included 1582 patients, of whom 1354 had conclusive diagnostic data from 2422 tubes and 1294 patients had data on intra-procedural pain. The mean age of included patients ranged from 28.8 to 34.7 years and their mean BMI from 20.8 to 26.1. The included patients had experienced on average 1.26 to 2.6 years of infertility. These basic study characteristics are summarized in Table 1. More detailed, technical characteristics of the diagnostic tests used per study are summarized in Table 2.

Diagnostic accuracy studies were assessed with the QUADAS-2C tool, with most studies demonstrating acceptable risk of bias and applicability (Table 3). The randomized controlled trial with intra-procedural pain as the primary outcome by Dreyer et al. [22] was assessed via the RoB 2 tool: low risk for the first, third and fourth domains and some concerns (intermediate risk) for the second and fifth domains. Overall, the study was evaluated as of intermediate risk of bias. Finally, the study by Serrano Gonzalez et al. [28], being a non-randomized study with intra-procedural pain as the primary outcome, was evaluated via the Newcastle–Ottawa Scale and received a grade of 7/9, indicating low risk to moderate risk of bias.

**Figure 2:**
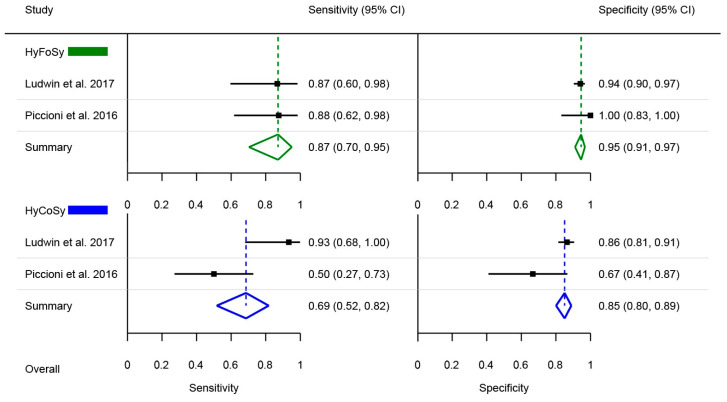
Forest plot of pooled sensitivity and specificity of HyFoSy compared to HyCoSy [24,25].

Regarding the HyFoSy and HyCoSy comparison, two studies used laparoscopic chromopertubation as the reference standard and thus were included in the analysis [24,25]. Heterogeneity statistics were as follows: for HyFoSy pooled sensitivity Cochran’s Q = 0.01, *p* = 0.940; I2 = 0%, for HyFoSy pooled specificity Cochran’s Q = 1.66, *p* = 0.198; I2 = 39.6%, for HyCoSy pooled sensitivity Cochran’s Q = 9.06, *p* = 0.003; I2 = 88.9% and for HyCoSy pooled specificity Cochran’s Q = 2.51, *p* = 0.113; I2 = 60.2%. Pooled sensitivity and specificity percentages were calculated based on the number of individual tubes assessed. Relative sensitivity and relative specificity ratios were calculated, along with *p*-values to assess statistical significance for each comparison. Pooled sensitivity for HyFoSy was 87% and for HyCoSy 69%, with a relative ratio of 0.79 (95% CI: 0.61–1.02, *p* = 0.074), a marginally statistically non-significant difference. Pooled specificity for HyFoSy was 95% and for HyCoSy 85%, with a relative ratio of 0.9 (95% CI: 0.85–0.95, *p* < 0.001), a statistically significant difference. The results are summarized in Figure 2.

The study by Lim et al. [23] also compared HyFoSy and HyCoSy, but without using a reference standard, rather by conducting cross-over testing in cases of obstruction. On initial testing, a higher proportion of tubal patency was diagnosed in the HyFoSy group compared to HyCoSy (70.0% vs. 40.0%, *p* = 0.01). For the cases of potential tubal obstructions based on the results of the initial assessment, more patients from the HyCoSy group required cross-over testing (16/20 patients: 80.0% vs. 9/20 patients: 45.0%, *p* = 0.02). Ultimately, 41.7% of tubes initially examined by HyCoSy and classified as obstructed or unexaminable were re-classified as patent with HyFoSy, whereas the corresponding percentage for the reverse (initial examination by HyFoSy and re-examination by HyCoSy) was 8.3%, a statistically significant difference (*p* = 0.03) [23].

**Figure 3:**
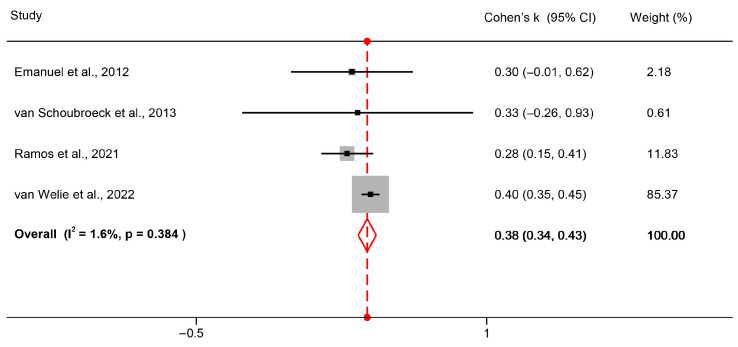
Forest plot of pooled Cohen’s k statistic for HyFoSy and HSG [20,21,26,27].

**Figure 4:**
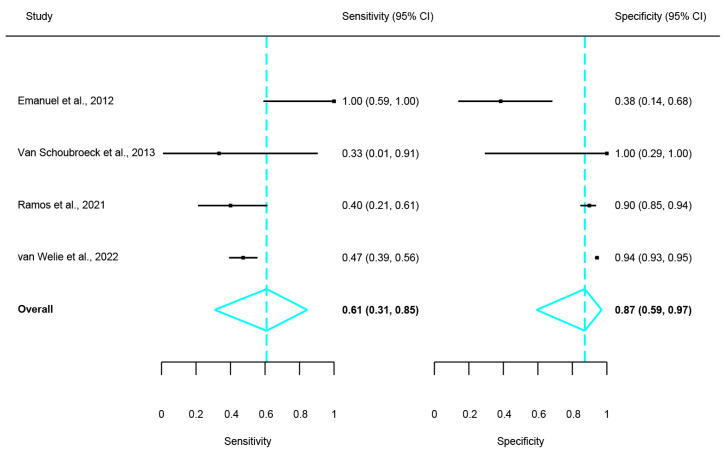
Forest plot of pooled sensitivity and specificity of HyFoSy when HSG was used as a reference standard [20,21,26,27].

Regarding the comparison of the diagnostic performance of HyFoSy versus HSG, none of the available studies used a reference standard and instead assessed the level of agreement between the two techniques. Four studies were applicable for this comparison [20,21,26,27]. Statistical heterogeneity parameters were as follows: Cochran’s Q = 3.05, *p* = 0.384 and I2 = 1.55%, overall indicative of non-significant statistical heterogeneity. The pooled Cohen’s k statistic resulting from the meta-analysis was 0.38, meaning that overall agreement between HyFoSy and HSG was fair, marginally moderate. These results are summarized in Figure 3. In order to further explore the association of HyFoSy and HSG, a diagnostic accuracy study was also conducted, utilizing the inter-method agreement data, with HSG considered the reference standard. The resulting sensitivity and specificity of HyFoSy when HSG was considered the reference standard was 61% and 87%, respectively. The results of this analysis are summarized on Figure 4.

**Figure 5:**
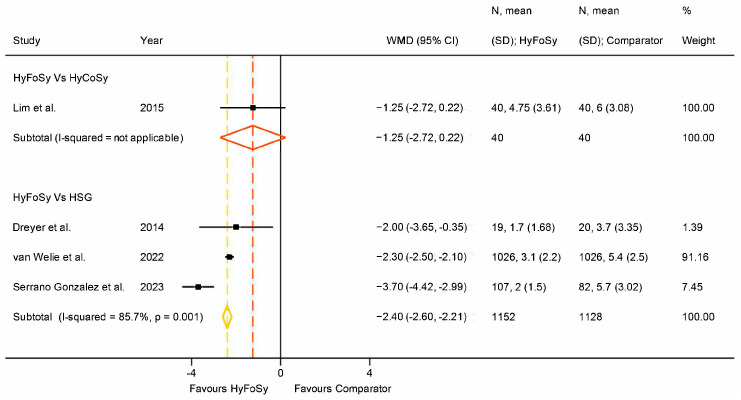
Forest plot of intraprocedural pain during HyFoSy, HyCoSy and HSG. Pain is graded on a 10-point scale, with higher values representing higher intensity [22,23,27,28].

Regarding intra-procedural pain, the patient-reported score from 0 (no pain at all) to 10 (the most severe pain ever experienced by the patient), recorded via visual analogue scale (VAS) immediately after testing, was utilized. Only the study by Lim et al. [23] compared HyFoSy and HyCoSy with regard to experienced pain, showing that HyFoSy was less painful by 1.25 VAS points. However, this difference was not statistically significant. With regard to the comparison of HyFoSy and HSG, data from multiple studies were available (I2 = 85.7%, *p* = 0.001) and showed that HyFoSy was again more tolerable, graded on average 2.4 VAS points lower than when compared to HSG, which was a statistically significant difference (*p* < 0.001). Data on intraprocedural pain are summarized on Figure 5. Ramos et al. [26] also assessed experienced pain, but the raw data were not meta-analyzable. Regardless, they showed that HSG was 6.5 times more likely to be evaluated as more painful compared to HyFoSy (OR = 6.57, CI 95% 3.11–13.89).

## 4. Discussion

In this systematic review we compared HyFoSy to the more widely used and established methods of HyCoSy and HSG. Based on the available data in the medical literature, we showed that HyFoSy possessed superior diagnostic specificity (95% versus 85%, *p* < 0.001) compared to HyCoSy, while also demonstrating superior diagnostic sensitivity, although marginally not statistically significant (87% versus 69%, *p* = 0.0367). The data from Lim et al. [23] also show a statistically significant tendency of HyFoSy to correctly diagnose patency compared to HyCoSy, during both the initial and subsequent assessment. In the comparison of HyFoSy and HSG, the two methods had a kappa value of 0.38, meaning that agreement between the two methods was fair to moderate according to Cohen’s initial interpretation [19], although more recent scholars interpret this level of agreement as minimal [29]. Overall, agreement between the two methods was not substantial enough to characterize HyFoSy as equal to HSG diagnostically. In a diagnostic accuracy analysis based on the same data and with HSG as the reference test, HyFoSy had poor sensitivity (61%) but increased specificity (87%). When the intra-procedural pain was assessed, HyFoSy and HyCoSy demonstrated a non-significant trend towards the former being more tolerable. However, when compared to HSG, HyFoSy was clearly superior, with a lower mean VAS score and a statistically significant weighted mean difference of 2.4 points (*p* < 0.001).

Tubal abnormalities have been diagnosed in approximately 35% of women experiencing infertility [30]. These abnormalities are usually the result of inflammatory conditions in the area, such as sexually transmitted infections, pelvic inflammatory disease, salpingitis and endometriosis, or disruption of normal tissue anatomy, such as history of pelvic injury or abdominal and/or pelvic surgery [31,32]. Given the increased incidence of tubal factor infertility, as well as the increased frequency of its underlying etiologic factors in women of reproductive age, tubal patency assessment has become routine during infertility work-up.

HyFoSy represents a feasible, well-tolerated and safe alternative for tubal patency assessment [33,34] to the established methods of HyCoSy and HSG. It utilizes a contrast medium which, following dilution with purified water and infusion into the uterine cavity, produces foam, which remains stable for approximately 5 min, providing visualization of the fallopian tubes [12,20]. The contrast medium, a mixture of glycerol, hydroxyethyl cellulose and purified water (ExEm gel^®^), has been shown to be safe and non-toxic, with no unexpected side-effects having been reported, in contrast to the widely reported allergic reactions to iodine-based contrast media, which are used in HSG [35]. Additionally, HyFoSy has been shown to exert a positive effect on fertility outcomes by itself, as studies have indicated a tendency for increased, either spontaneous or ART-assisted conception and pregnancy rates after HyFoSy, most likely mediated by the “tubal flushing” effect [34,36,37,38].

Despite the additional advantages, diagnostic efficacy remains amongst the most important factors. With regard to diagnostic sensitivity, we demonstrated that HyFoSy was equivalent (higher sensitivity but marginally non-significant difference) to HyCoSy, the ultrasonographic alternative. Additionally, HyFoSy demonstrated significantly higher diagnostic specificity than HyCoSy. This translates into improved ability in detecting and verifying patency, which was also corroborated by the findings of Lim et al. [23]. The investigators showed that when each of the two ultrasonographic methods were used to re-evaluate abnormal results produced on initial testing by the other, HyFoSy was significantly more likely to re-classify obstructed tubes as patent, while the same was not true for HyCoSy. The investigators concluded that HyFoSy offers an increased patency detection rate, meaning increased specificity, as was shown in our analysis as well, while also accurately recognizing tubal obstruction [23].

When the diagnostic agreement of HyFoSy and HSG was examined, the methods showed minimal to moderate agreement, depending on the applied interpretation method. Regardless, this level of agreement implies considerable differences between the two methods. Given the fact that HSG is a radioscopic method, it may be considered less operator-dependent and thus more objective. Following up on this assertion, when we examined the diagnostic performance of HyFoSy with the results of HSG as a reference standard, HyFoSy demonstrated low sensitivity but relatively high specificity, indicating that it is better suited to confirm tubal patency in healthy fallopian tubes, rather than reliably confirming the presence of obstruction and pathology.

With regard to intra-procedural pain, HyFoSy and HyCoSy demonstrated no statistically significant differences in the single study that compared them in this context [23]. This was not the case for the HyFoSy and HSG comparison, with the former being evaluated as significantly more tolerable with regard to pain compared to the latter, with an average difference of 2.4 points on a 10-point scale. This difference is, in the authors’ opinion, not only statistically significant, but also clinically meaningful. This is due to the size of the difference, representing nearly a quarter of the scale, and due to the high frequency of tubal patency assessment in the context of assisted reproduction. Tubal patency assessment is nowadays considered a baseline test, recommended to all patients prior to treatment planning and it is a test that may be repeated after some time has elapsed or if the couple visits a different clinic. Therefore, the prospect of a nearly mandatory test that may be repeated would be considerably more tolerable by patients if it were significantly less painful, as is the case with HyFoSy. This conclusion is also corroborated by Serrano Gonzalez et al. [28], who, in addition to intra-procedural pain, also examined patient anxiety after the examination. This is a very important but frequently overlooked factor. The investigators utilized the State Trait Anxiety Inventory (STAI) questionnaire and compared the resulting scores between the two methods. HyFoSy had a median State-STAI score of 10, while HSG had a score of 18, a statistically significant difference (*p* < 0.001) in anxiety associated with the examination itself. When basal anxiety was examined, only a small difference was detected (HyFoSy median Trait-STAI score: 13 versus HSG median Trait-STAI score: 15, *p* = 0.044), indicating that the vast majority of observed anxiety could be solely attributed to the applied diagnostic method and not any idiosyncratic differences between the two patient groups [28]. This conclusion is supported by other similar studies as well [22,39].

This is, to our knowledge, the first comparative systematic review and meta-analysis of the diagnostic efficacy and tolerability of HyFoSy compared to HyCoSy and HSG. While we managed to prove HyFoSy’s diagnostic superiority compared to HyCoSy, no such feat was possible in the comparison with HSG, as the two methods showed inadequate levels of concordance. However, with HSG acting as a reference standard, HyFoSy demonstrated increased diagnostic specificity and, as has been reported in other studies, tubal patency is more reliably diagnosed than occlusion, particularly with ultrasonographic assessment methods [5,40]. Additionally, as we demonstrated, HyFoSy is an exceedingly more tolerable assessment test for the patient compared to HSG. Furthermore, HyFoSy confers several practical advantages such as the potential to be performed within the fertility clinic or the gynecologist’s office, with the available ultrasound equipment, requiring no specialized radiographer or a radiologist to perform and thus being less expensive. This practicality may translate to additional benefits to the patient, as they may avoid a more painful and stressful procedure that involves ionizing radiation, with potentially lower overall costs, both due to reduced cost of the test itself, as well as due to improved logistics (no need to leave the fertility clinic, one-stop visit, etc.). However, there are also several challenges that need to be addressed, which include primarily the issue of operator dependence for HyFoSy results, which is due to the use of ultrasound, a more inherently subjective method than HSG. However, the use of three-dimensional ultrasound has the potential to address this issue, having demonstrated improved reproducibility of findings and inter-operator concordance in other fields of reproductive medicine [41,42]. Another potential challenge in the application of HyFoSy in clinical practice could potentially be the availability of the contrast medium, as it is patented and production may be limited. However, as the application of HyFoSy expands, supply will likely rise to meet the increasing demand, resolving this issue over time. The main characteristics of each of the three diagnostic modalities, along with advantages and disadvantages based on the findings of the present study and the authors’ conclusions, are summarized in Table 4.

Based on all the data presented, two patient workflow scenarios could be considered. For the average fertility clinic patient with no prior tubal patency assessment results, or with normal but outdated results, HyFoSy may be feasibly applied as a first-line diagnostic test, instead of HyCoSy due to superior diagnostic performance and instead of HSG due to practical advantages (less painful, conveniently available within the fertility clinic, etc.) and the increased diagnostic specificity, meaning the ability to confirm patency. HSG may be utilized as a second-line test, for cases where patency was not proven by first-line testing, or where visualization difficulties exist [43]. Finally, for fertility patients with risk factors for tubal impairment (history of pelvic inflammatory disease, endometriosis, etc.) or with previous abnormal but outdated results, HSG could be considered as a first-line test due to the poorer performance of HyFoSy in diagnostic sensitivity (in the definitive confirmation of obstruction).

There are certain limitations to this study that should be acknowledged. Firstly, the small number of available comparative studies on HyFoSy. Secondly, there was a lack of studies that used a reference standard, with only two being available for the HyFoSy–HyCoSy comparison and none for those comparing HyFoSy to HSG. This fact limits the applicability and reliability of the conclusions, for the former due to the limited number of studies and for the latter due to the use of agreement statistics only (without a reference standard). Furthermore, the small number of available studies limits the reliability of findings, as the introduction of newer studies could potentially lead to changes in the effects and trends measured in the present analysis. Finally, despite the similarities in design of the included studies, there was a degree of methodological and statistical heterogeneity, which may have introduced bias in our results.

## 5. Conclusions

Tubal factor pathology is very frequently a contributing factor in multiple cases of infertility. Accurate, safe and tolerable fallopian tube patency assessment is a key component of every infertility work-up algorithm. HyFoSy is a promising method, which was shown to be diagnostically superior to the other ultrasonographic alternative, namely HyCoSy, although this was based on findings from only two studies with a reference standard. HyFoSy showed moderate concordance with HSG results (based on studies without a reference standard); however, being less painful and more reliable in diagnosing patency, it may be applicable as a first line diagnostic test, with HSG being used to verify abnormal results. These findings should however be interpreted cautiously, as the scarcity of comparative studies with a reference standard prevents direct comparison of each method’s diagnostic efficacy. Additional, more rigorously designed comparative studies that use a reference standard are required in order to definitively define the role that HyFoSy should play during infertility investigation.

## Figures and Tables

**Table 1:** Summary of the baseline demographic characteristics of the participants of the included studies.

Study	Year	Country	Recruitment Period	Location	Sample	Mean Age ± SD (y)	Mean BMI ± SD	Mean Infertility Duration ± SD (y)	Index Test	Comparator	Reference Test	Outcomes
Emanuel et al. [20]	2012	The Netherlands	In 2010	Single-centre	10	34 ± 3.8	24.5 ± 4.8	2.2 ± 1.4	HyFoSy	HSG	N/A	Inter-method agreement
Van Schoubroeck et al. [21]	2013	Belgium	N/S	Single-centre	3	30.5 ± 4.5	N/S	N/S	HyFoSy	HSG	Laparoscopic chromo-pertubation	Inter-method agreement
Dreyer et al. [22]	2014	The Netherlands	Jan 2013–Sept 2013	Two-centre	39	33 ± 4.8	20.8 ± 3.7	1.26 ± 0.67	HyFoSy	HSG	N/A	Procedure pain
Lim et al. [23]	2015	Singapore	April 2014–Dec 2014	Single-centre	40	28.8 ± 9.1	N/S	1.86 ± 1.5	HyFoSy	HyCoSy	N/A	Inter-method agreement, procedure pain
Piccioni et al. [24]	2016	Italy	Sep 2014–Oct 2015	Single-centre	37	34 ± 0.5	26.1 ± 1.2	2.4 ± 0.6	HyFoSy	HyCoSy	Laparoscopic chromo-pertubation	Diagnostic accuracy
Ludwin et al. [25]	2017	Poland	Nov 2013–July 2015	Three-centre	132	32.3 ± 4.3	22.2 ± 3.66	2.6 ± 1.8	HyFoSy	HyCoSy	Laparoscopic chromo-pertubation	Diagnostic accuracy
Ramos et al. [26]	2021	Spain	June 2013–Feb 2017	Single-centre	106	34.71 ± 3.68	24.11 ± 4.54	2.30 ± 1.70	HyFoSy	HSG	N/A	Inter-method agreement
Van Welie et al. [27]	2022	The Netherlands	N/S	26-centre	1026	33.0 ± 4.45	23.7 ± 4.2	1.7 ± 0.7	HyFoSy	HSG	N/A	Inter-method agreement, procedure pain
Serrano Gonzalez et al. [28]	2023	Spain	Sep 2017–Oct 2018	Two-centre	189	34.6 ± 3.2	N/S	N/S	HyFoSy	HSG	N/A	Procedure pain

N/A: not applicable.

**Table 2:** Summary of the characteristics of the diagnostic modalities used in each of the included studies.

	Index Test (HyFoSy)							Comparator Test						
Study	Model	Frequency	Timing	Contrast Type	Contrast/Water Ratio	Contrast Volume, Infusion Rate	Catheter	Comparator	Model	Contrast Agent	Contrast/Air ratio	Contrast Volume, Infusion Rate	Catheter	No of Radiographs
Emanuel et al. [20]	N/S	N/S	N/S	ExEm-gel^®^	10 mL/10 mL	20 mL, NS	GIS cervical balloon-less catheter	HSG	N/S	N/S	N/A	N/S	N/S	N/S
Van Schoubroeck et al. [21]	Voluson E8	6–12 MHz	N/S	ExEm-gel^®^	10 mL/10 mL	2–5 mL, NS	2 mm pediatric Foley balloon	HSG	N/S	N/S	N/S	N/S	N/S	N/S
Dreyer et al. [22]	EnVisor	N/S	End of menses−14th day of the cycle	ExEm-gel^®^	10 mL/10 mL	10 mL, 1.67 mL/min	Cervical balloon-less catheter	HSG	AXIOM Iconos R 200	Telebrix Hystero, Lipiodol Ultra	N/A	10 mL, 1.67 mL/min	Hysterophore	6–8
Lim et al. [23]	Voluson E8	6–12 MHz	6–12th day of the cycle	ExEm-gel^®^	10 mL/10 mL	20 mL, NS	Cervical balloon-less catheter	HyFoSy	Same as HyFosy	Saline solution	N/S	20 mL, N/S	No. 5 pediatric Foley catheter	N/A
Piccioni et al. [24]	Accuvix A30	5–9 MHz	7–13th day of the cycle	ExEm-gel^®^	10 mL/10 mL	20 mL	cervical balloon-less applicator	HyCoSy	Same as HyFosy	Saline solution	15 mL/5 mL	20 mL, N/S	5-French balloon catheter	N/A
Ludwin et al. [25]	Voluson E8 Expert	5–9 MHz	5–12th day of the cycle	ExEm-gel^®^	10 mL/10 mL	20 mL	5-French balloon catheter	HyCoSy	Same as HyFosy	Saline solution	10 mL/10 mL	20–40 mL, N/S	5-French balloon catheter	N/A
Ramos et al. [26]	Voluson 730 proV	4–9 MHz	N/S	ExEm-gel^®^	10 mL/10 mL	5 mL	GIS cervical balloon-less catheter *	HSG	N/S	N/S	N/A	N/S	N/S	N/S
Van Welie et al. [27]	Varied per center	Varied per center	2–14 day of the cycle	ExEm-gel^®^	5 mL/5 mL	5–10 mL	GIS cervical balloon-less catheter	HSG	N/S	Varied per center	N/A	5–10 mL, N/S	vacuum cervical cup, hysterophore, balloon catheter	6–8
Serrano Gonzalez et al. [28]	Voluson 730 Pro	6–12 MHz	6–12 day of the cycle	ExEm-gel^®^	N/S	3–10 mL	Unomedical CH6, 6-Fr Kitazato balloon-less, Foerster/Pozzi clamp for assistance	HSG	Siemens C-shaped arc	Visipaque TM 270 mg/mL	N/A	Max 10 mL, N/S	5-French balloon catheter	N/S

*: other catheters were also used in case of difficulties, N/S: not specified, N/A: not applicable.

**Table 3:** Summary of QUADAS-2 findings per domain.

Study	Year	Risk of Bias (QUADAS-2)				Applicability (QUADAS-2)			Risk of Bias (QUADAS-2C)			
		P.S.	I.T.	R.S.	F.T.	P.S.	I.T.	R.S.	P.S.	I.T.	R.S.	F.T.
Emanuel et al., 2012 [20]	HyFoSy	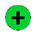	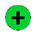	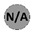	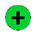	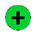	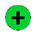	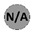	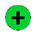	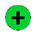	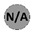	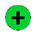
	HSG	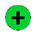	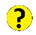	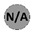	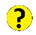	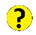	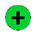	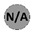				
van Schoubroeck et al., 2013 [21]	HyFoSy	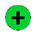	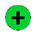	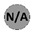	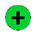	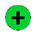	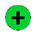	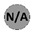	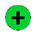	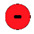	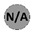	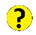
	HSG	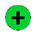	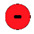	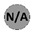	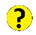	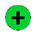	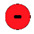	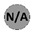				
Lim et al., 2015 [23]	HyFoSy	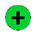	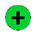	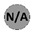	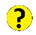	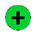	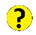	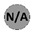	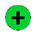	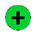	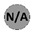	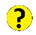
	HyCoSy	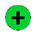	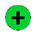	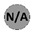	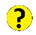	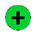	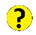	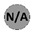				
Piccioni et al., 2016 [24]	HyFoSy	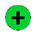	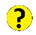	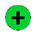	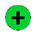	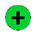	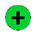	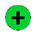	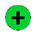	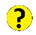	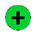	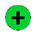
	HyCoSy	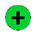	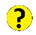	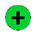	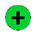	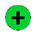	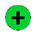	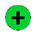				
Ludwin et al., 2017 [25]	HyFoSy	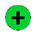	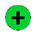	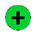	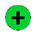	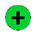	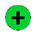	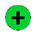	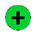	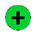	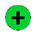	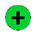
	HyCoSy	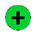	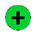	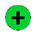	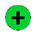	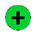	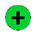	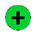				
Ramos et al., 2021 [26]	HyFoSy	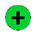	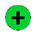	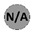	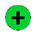	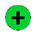	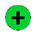	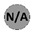	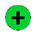	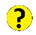	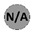	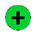
	HSG	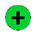	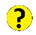	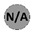	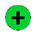	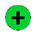	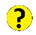	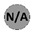				
van Welie et al., 2022 [27]	HyFoSy	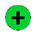	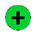	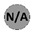	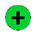	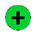	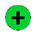	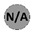	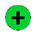	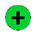	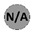	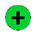
	HSG	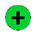	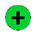	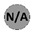	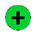	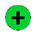	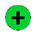	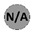				

P.S.: Patient Selection, I.T.: Index Test, R.S.: Reference Standard, F.T.: Flow and Timing, +: low risk, −: high risk, ?: unclear, N/A: not applicable.

**Table 4:** Summary of the characteristics, advantages and disadvantages per tubal patency assessment test.

		HyFoSy	HyCoSy	HSG
Characteristics	Sensitivity	Same as HyCoSy (better performance than HyFoSy based on the study by Lim et al. 2015 [23]) Low agreement with HSG, poor concordance for true positive cases	Same as HyFoSy (poorer performance than HyFoSy based on the study by Lim et al. 2015 [23])	Low agreement with HyFoSy and low concordance for true positive cases
	Specificity	Higher than HyCoSy Low agreement with HSG, but high concordance for true negative cases	Lower than HyCoSy	Low agreement with HyFoSy, but high concordance for true negative cases
	Procedure pain	Same as HyCoSy Significantly lower than HSG	Same as HyFoSy	Higher than HyFoSy
	Practicality	Same as HyCoSy Higher than HSG	Same as HyFoSy	Lower than HyFoSy
Summary of findings	Advantages	High specificity (reliable confirmation of patency) Low pain Available at outpatient level Performed by Fertility Specialist Radiation-free	Low pain Available at outpatient level Performed by Fertility Specialist Radiation-free	Best diagnostic performance High sensitivity Reproducible results
	Disadvantages	Low sensitivity (reliable confirmation of obstruction) Limited reproducibility of results	Low specificity Limited reproducibility of results	High pain Available only at Radiology Centre Most commonly performed by Radiologist Radiation and iodine-based contrast

## Data Availability

All generated data are provided within the manuscript.

## Abbreviations

The following abbreviations are used in this manuscript:

HyFoSy	Hysterosalpingo-Foam Sonography
HyCoSy	Hysterosalpingo-Contrast Sonography
HSG	Hysterosalpingography
